# Deep-Sequencing Identification of MicroRNA Biomarkers in Serum Exosomes for Early Pig Pregnancy

**DOI:** 10.3389/fgene.2020.00536

**Published:** 2020-05-26

**Authors:** Chen Zhou, Gengyuan Cai, Fanming Meng, Zhiqian Xu, Yanjuan He, Qun Hu, Enqin Zheng, Sixiu Huang, Zheng Xu, Ting Gu, Bin Hu, Zhenfang Wu, Linjun Hong

**Affiliations:** ^1^National Engineering Research Center for Breeding Swine Industry, College of Animal Science, South China Agricultural University, Guangzhou, China; ^2^Guangdong Provincial Key Laboratory of Agro-Animal Genomics and Molecular Breeding, College of Animal Science, South China Agricultural University, Guangzhou, China; ^3^Lingnan Guangdong Laboratory of Modern Agriculture, Guangzhou, China; ^4^Institute of Animal Science, Guangdong Academy of Agricultural Sciences, Guangzhou, China

**Keywords:** microRNA, early pregnancy, exosomes, sequencing, pig

## Abstract

Early pregnancy diagnosis in sows can significantly improve the efficiency of pig industry. Exosomes are membrane-covered nanovesicles that can transport microRNAs (miRNAs) and other molecular signals between cells. In other species, serum exosome-derived miRNAs can serve as good biomarkers of diseases and different physiological states, including pregnancy status. We hypothesized that circulating exosome-derived miRNAs might be used to differentiate the pregnancy status as early as several days after insemination in pigs. To test this hypothesis, we randomly assigned pigs for artificial insemination with fertile or dead semen (control group). Serum samples were obtained from pregnant pigs on days 9, 12, and 15 after insemination and from non-pregnant pigs on days 0, 9, 12, and 15 after insemination. Exosomes were isolated for RNA extraction. The exosomal RNA samples from pigs on day 9 of the estrus cycle and pregnancy were used for small-RNA sequencing. A total 321 miRNAs were identified in all samples. Twenty eight differentially abundant miRNAs were identified between the pregnant and control groups. miRNAs with | log2 (fold change)| > 2 from sequencing results were selected for validation by quantitative reverse-transcription-polymerase chain reaction (RT-qPCR) in larger samples. Finally two upregulated miRNAs (miR-92b-3p and miR-17-5p) in the pregnant groups (on days 9, 12, and 15 of pregnancy) were confirmed by RT-qPCR. In summary, we have successfully identified circulating exosomal miRNA profiles in the serum of pigs in early pregnancy. miR-92b-3p and miR-17-5p could be used as potential circulating biomarkers for early pregnancy diagnosis.

## Introduction

Early pregnancy diagnosis in sows is an important technique to improve the breeding efficiency of the swine industry. Sows that fail to conceive after insemination and cannot be detected with pregnancy at an early period could cause large economic loss. To date, several methods have been developed to detect pregnancy in pigs after fertilization, including the signs of non-return to estrus cycle and the increase in ventral abdomen during pregnancy combined with ultrasonic diagnosis. Pregnancy status can also be detected by concentrations of progesterone or estrogen in blood, urine, or feces (Resnis et al., 2000; Kauffold and Althouse, 2007; Rousian et al., 2009; Agnieszka, 2011). However, these methods can only detect the pregnancy status as early as days 25–30, and no currently available method diagnoses pregnancy before 25 days after fertilization.

Exosomes, as a new medium for transporting signaling molecules, play an important role in cell communication. Exosomes are extracellular vesicles of phospholipid bilayer with sizes of 30–150 nm, these vesicles could carry proteins, lipids, DNA, RNA, and other non-coding RNAs (ncRNAs), including microRNA (miRNAs) (Jeppesen et al., 2019). Exosomal miRNAs that are secreted into bio fluids, such as urine, serum, tears, plasma, and gastric juice, may not be degraded by RNases (Gilad et al., 2008). Additionally, circulating miRNA expression profile features higher accuracy exact than circulating mRNA expression profiles (Lu et al., 2005). Increasing evidence indicates that miRNA expression profiles measured in bio fluids may reflect biological processes in different organisms (Kuner et al., 2013). For instance, more than eight types of cancer have been proven to be related with specific exosome-derived miRNAs that are currently used as biomarkers for cancer prediction (Thind and Wilson, 2016). Interestingly, the specific circulating miRNAs detected in maternal plasma during pregnancy could be associated with pregnancy status. Pohler et al. (2017) observed that extracellular vesicle (i.e., exosomes and microvesicles)-derived miRNAs in the serum can be used to distinguish the pregnancy status in cows before day 24 of pregnancy (Pohler et al., 2017). Therefore, the detection of maternal blood circulating miRNAs shows potential to become a promising non-invasive prenatal diagnostic method in animals (Gu et al., 2019).

In this study, we hypothesized that specific circulating exosome-derived miRNAs may be considered candidate diagnostic markers for early pregnancy of sows. To test this hypothesis, we isolated and purified exosomes in the serum of pregnant and non-pregnant pigs through ultracentrifugation and observed and analyzed the results by transmission electron microscopy and immunoblotting protein analysis. Then, exosomal miRNAs were detected by small-RNA sequencing, and the differentially expressed miRNAs (DE miRNAs) were identified by bioinformatics analysis. Finally, DE miRNAs were further confirmed by RT-qPCR analysis in larger sample set.

## Materials and Methods

### Sample Collection

For RNA sequencing analysis, six healthy Yorkshire gilts were obtained from Wen’s Foodstuffs Group Co Ltd (Yunfu, China). The gilts were either artificially inseminated with fresh semen (pregnant group) or sham-inseminated using killed semen from the same source (non-pregnant group) 12 h after the onset of estrus (day 0) and again 24 h later. Blood samples (10 mL) were collected from the ear vein on day 9 (*n* = 3) of the estrus cycle (C9, non-pregnant gilts) and day 9 of pregnancy (P9, *n* = 3). Then, sera were obtained by low-speed centrifugation. Pregnancy was confirmed by the presence of conceptuses in uterine flushing after the gilts were slaughtered at a local slaughterhouse.

For the qPCR experiment, serum samples were collected from another group of Yorkshire sows (parity 2–3) on days 0 (C0, *n* = 8), 9 (C9, *n* = 8), 12 (C12, *n* = 8), and 15 (C15, *n* = 8) of the estrus cycle and days 9 (P9, *n* = 8), 12 (P12, *n* = 8), and 15 (P15, *n* = 8) of pregnancy. The treatments and methods were the same as those applied for RNA sequencing experiment. In this experiment, pregnancy was confirmed after the delivery of pigs.

### Isolation of Exosomes From Serum

Exosomes were isolated from the sera as previously described (Thery et al., 2006). Briefly, the separated sera were centrifuged at 2,000 × g for 20 min to remove fragments and subsequently subjected to centrifugations at 11,000 × g for 30 min to further eliminate cell debris. The supernatant obtained in the previous step was filtered through a Millex GP 0.22 μm filter. Finally, the supernatant was ultracentrifuged twice at 111,000 × g for 2 h using an SW41T rotor (Beckman Coulter, United States). All centrifugation steps were performed at 4°C. The pellet was dissolved in phosphate-buffered saline (PBS) and stored at −80°C until analysis.

### Transmission Electron Microscopy (TEM) Analysis

The exosomes (8 μL) were placed on a copper grid and negatively stained with equal quantities of saturated aqueous uranyl acetate for 3 min at room temperature. Next, the samples were dried naturally. Finally, the images of exosomes were captured under a transmission electron microscope (FEI Talos F200S) at 80 kV.

### Nanoparticle Tracking Analysis (NTA)

The particle size and concentration of exosomes were measured by NTA using a ZetaView PMX 110 (Particle Metrix, Germany) and the corresponding software ZetaView 8.04.02. The exosomes were diluted 4,000 times with PBS to measure the particle size and concentration. NTA was performed on 11 positions, and the measurement results were recorded.

### Western Blotting

The exosomes were treated with radioimmunoprecipitation assay (RIPA) lysis buffer (CWBIO, RIPA = 1:100) to isolate the total protein. Micro BCA Protein Assay Kit (CWBIO) was used to measure the protein concentration. The exosomal proteins were heat-denatured and separated by 12% sodium dodecyl sulfate polyacrylamide gel electrophoresis. The exosomal proteins were transferred to a polyvinylidene fluoride membrane (Millipore, United States) and blocked with 6% skim milk powder for 2.5 h at room temperature. Then, the proteins were incubated with the anti-CD9/CD63 (ab2215/ab59479, Abcam, United Kingdom) and horseradish peroxidase-conjugated goat-anti-mouse IgG (Abcam, United Kingdom). Blots were developed using the BeyoECL Moon Chemiluminescence Development Kit (Beyotime, China).

### Illumina Small-RNA Sequencing and Data Analysis

Total RNA was isolated from the serum exosomes using the exoRNeasy Serum/Plasma Maxi Isolation Kit (Qiagen, Germany) in accordance with the manufacturer’s instructions. RNA was re-suspended in 14 μL RNase-free water and used immediately or frozen at −80°C. Small-RNA cDNA libraries were generated using NEBNext Multiplex Small RNA Library Prep Set for Illumina^®^ (NEB, United States) following the manufacturer’s recommendations. The library preparations were sequenced on an Illumina Novaseq 6000 platform, and 50 bp single-end reads were generated. The small RNA tags were mapped to the reference sequence by Bowtie (Langmead et al., 2009) without mismatch to analyze their expression and distribution on the reference. The miRNA expression levels were estimated by transcript per million (Zhou et al., 2010). Differential expression analysis of two conditions was performed using the DEGseq (2010) R package. *P*-values were converted to adjusted *P*-values using the Benjamini–Hochberg method. The adjusted *P* < 0.05 and | log2 (fold change)| > 1 were set as the thresholds for significantly differential expression. All the procedures for small-RNA library preparation and sequencing analysis were accomplished by Novogene (Beijing, China).

### Validation of MiRNA Expression via RT-qPCR

Total RNA was isolated from the serum exosomes using the exoRNeasy Serum/Plasma Maxi Isolation Kit (Qiagen, Germany) according to the manufacturer’s protocol. Isolated RNAs were validated by RT-qPCR on the enlarged serum samples (*n* = 8 per group). cDNA was generated using the TransScript^®^ miRNA First-Strand cDNA Synthesis Super Mix (TransGen, China) and diluted for use in 10 μL qPCR reactions using Thermo SYBR Green kits in a QuantStudio 7 Flex Real-Time PCR System (life, United States). Thermocycler settings were: 95°C for 5 min, followed by 40 cycles of 95°C for 20 s, 60°C for 15 s, and 72°C for 20 s, followed by 3 min at 72°C. The primers used in the validation assays were designed on the basis of miRNA mature sequence and listed in Supplementary Table S1. The exogenously added spike-in cel-miR-39 (Qiagen, Germany) was used as the reference (Salvoza et al., 2016). The relative expression levels were calculated using the 2^–ΔΔ*Ct*^ method and analyzed using SPSS version 21.0 (SPSS, Inc., Chicago, IL, United States). Paired *t*-tests and two-way analyses of variance were performed for analysis. *P* < 0.05 indicated a statistically significant difference.

### Bioinformatics Analysis

Target prediction for the DE miRNAs validated by qPCR was conducted using TargetScan 7.2 (www.targetscan.org/vert_72/). Functional analyses of Kyoto Encyclopedia of Genes and Genomes (KEGG) pathways^1^ was performed by using KOBAS.^2^ For statistical significance, the binomial test using a threshold corrected P < 0.05 was applied. To increase clarity of the results, all canonical pathways associated with toxicity, cancer and xenobiotic metabolism were removed as previously described (Reliszko et al., 2017).

## Results

### Isolation and Identification of Serum Exosomes

**FIGURE 1:**
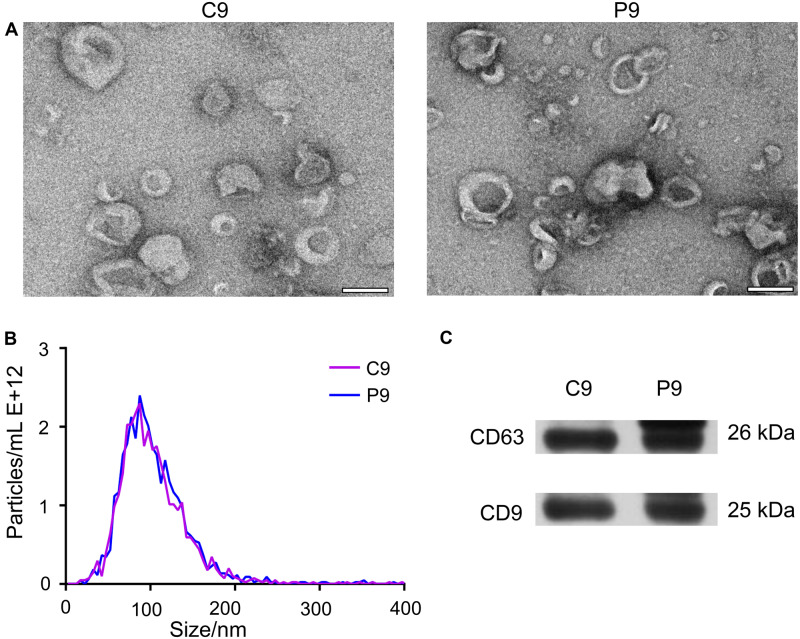
Characteristics of exosomes during early pregnancy. **(A)** TEM revealed vesicles with sizes of about 100 nm, consistent with those of exosomes. Representative TEM micrographs of exosomes derived from sera on day 9 (*n* = 3) of the estrus cycle (C9) and on day 9 (P9) of pregnancy. Scale bar = 100 nm. **(B)** NTA showed that most of vesicles were 30–150 nm in diameter. The profile confirmed the size measurement from TEM micrographs. **(C)** Western blot showing the presence of exosomal markers CD63 and CD9 in serum-derived exosomes from cyclic and pregnant pigs.

The isolated vesicles from the serum were discovered by TEM. Most of the vesicles measured 30–150 nm in diameter, consistent with the size of exosomes (Figure 1A). Nanoparticle tracking analysis showed that more than 89.4% of the vesicles were 30–150 nm in diameter (Figure 1B). This size range is similar to that detected by TEM and further confirms the identity of these vesicles as exosomes. Western blot analysis revealed that the isolated vesicles from the serum samples were positive for CD9 and CD63 proteins (Figure 1C), which are well-known exosomal protein markers.

### Small-RNA Sequencing of Serum Exosomes

**FIGURE 2:**
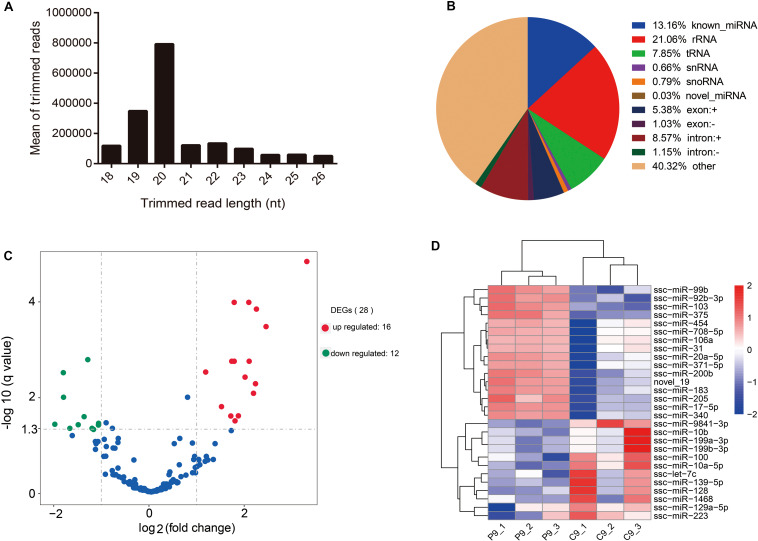
Overview of small RNA-Seq data. **(A)** Length distribution of read counts (after trimming) from Illumina sequencing of exosome samples (nt = nucleotides). **(B)** Relative abundance of different RNA species sequenced in porcine serum exosomes. **(C)** Volcano plots of differentially expressed miRNAs. X-axis denotes fold change (log_2_); Y-axis refers to the q value (−log_10_). **(D)** Hierarchical clustering of DE miRNAs.

Six small RNA libraries were generated from pregnant pigs on day 9 after insemination (*n* = 3) and from non-pregnant pigs on day 9 after insemination (*n* = 3). Altogether, we obtained 85,300,520 (accounting for 98.545% of the total reads) clean reads. On the average, 78.33% of the total clean reads comprised 19–22 nucleotides (nt) in length (Figure 2A). sRNA annotation showed that the proportions of known miRNAs, rRNA, tRNAs, snRNAs, snoRNAs, novel miRNAs, exonic RNAs, and intronic RNAs in serum-derived exosomes reached 13.16, 21.06, 7.85, 0.66, 0.79, 0.03, 6.41, and 9.72%, respectively (Figure 2B). A total of 28 DE miRNAs were identified between the pregnant and non-pregnant groups; 16 miRNAs were up-regulated, and 12 miRNAs were down-regulated under the pregnancy status (Figures 2C,D and Supplementary Table S2).

### RT-qPCR Assay of Pregnant and Non-pregnant Animals

**FIGURE 3:**
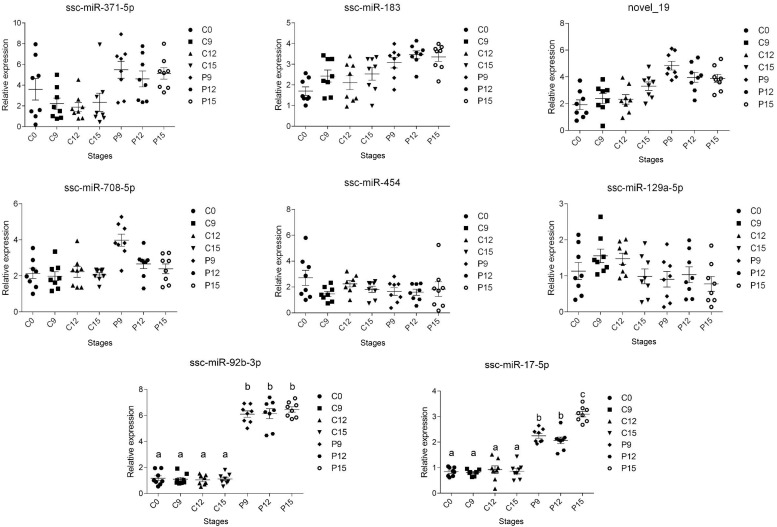
Two miRNAs validated by RT-qPCR in an independent group of pigs. RT-qPCR data plots (with mean ± standard error of the mean) obtained from an independent group of sows proving the increased circulating exosomal miRNAs (miR-92b-3p and miR-17-5p) levels during early pregnancy. The different superscript alphanumeric characters indicate statistically significant difference at *P* < 0.05.

The expressions of excellent biomarkers for early pregnancy should be persistently higher or lower during early pregnancy. To identify the biomarkers with a high credibility during early pregnancy, DE miRNAs with | log2 (fold change)| > 2 from sequencing results (8 miRNAs) were selected for validation by RT-qPCR in an independent group of sows (*n* = 8 per day). Results showed that compared to the non-pregnant group, the expression of miR-92b-3p and miR-17-5p was significantly increased (*P* < 0.05) as early as day 9 of pregnancy and kept at a high level during the early period of pregnancy (from days 9 to 15 of pregnancy) (Figure 3).

### Prediction of Target Genes and Functional Analysis

**FIGURE 4:**
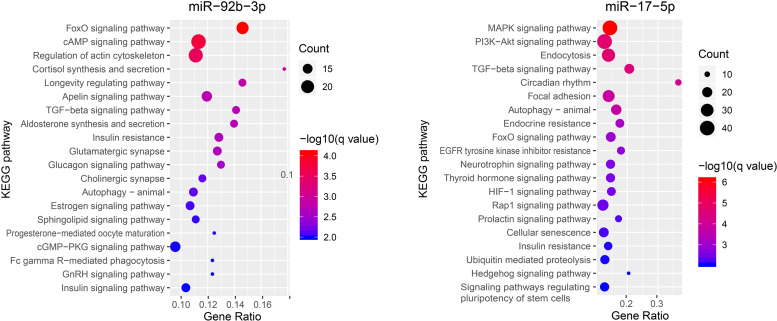
The KEGG pathway enrichment analysis on target genes of miR-92b-3p and miR-17-5p.

Since our qPCR experiment confirmed that miR-92b-3p and miR-17-5p were significantly increased in circulating exosomes of pregnant groups, we decided to perform functional analysis for these two miRNAs. A total of 1,041 genes for miR-92b-3p and 1,385 genes for miR-17-5p were revealed by TargetScan. KEGG enrichment analysis of the predicted genes for the miR-92b-3p indicated that 7 enriched pathways in the top 20 enriched pathways were related to hormone metabolism. These included Cortisol synthesis and secretion, Aldosterone synthesis and secretion, Insulin resistance, Estrogen signaling pathway, Progesterone-mediated oocyte maturation, GnRH signaling pathway and Insulin signaling pathway. While, pathways related to the targets for the miR-17-5p were mainly enriched in MAPK signaling pathway, PI3K-Akt signaling pathway, Endocytosis, TGF-beta signaling pathway and Circadian rhythm (Figure 4 and Supplementary Table S3).

## Discussion

Exosomal miRNAs in blood serum have been largely investigated as novel non-invasive biomarkers for different forms of cancer and physiological states including abnormal gestation (Pohler et al., 2017). In the current study, we hypothesized that specific circulating exosomal miRNAs can be used as diagnostic markers to differentiate the early pregnancy status of sows. For the first time, we applied several molecular biological techniques that allowed the detection of exosomal miRNAs in maternal serum of pigs with different pregnant statuses. As a unique set of miRNAs, miR-92b-3p and miR-17-5p can already be observed in the circulation of pigs as early as day 9 of pregnancy.

In our study, two miRNAs (miR-92b-3p and miR-17-5p) were validated in an independent group of sows (*n* = 8 per group) by qPCR. Results demonstrated that levels of exosomal miR-92b-3p and miR-17-5p were significantly increased in the maternal serum collected as early as day 9 of a pregnancy in pigs. These results are especially important because the capability to detect circulating miRNAs in the maternal serum may be used to create a new method for monitoring pregnancy stages in pigs as early as possible and long before day 25, when validation can be defined by ultrasonography (Martinatbotté et al., 2000).

miRNAs are small non-coding RNA molecules that post-transcriptionally regulate gene expression by base pairing (Wu et al., 2006). They have been hypothesized to play roles as the “oldest” hormones secreted into the serum and affect various target tissues in the body (Cortez et al., 2011). For miR-17-5p, many KEGG pathways that are crucial for reproduction were significantly enriched, including MAPK, PI3K-Akt, and TGF-beta signaling pathways, which were in the top 5 enriched pathways. Of note, MAPK signaling pathway was involved in conceptus implantation in pigs, and the activation of this pathway could increase the proliferation of porcine endometrial cells (Lim et al., 2018). In addition, the PI3K-Akt signaling pathway is also related to proliferation of endometrial cells and conceptus implantation in goat and mouse (Chang et al., 2018; Zhang et al., 2018). TGF-beta signaling pathway was demonstrated to regulate multifaceted reproductive processes, including the embryo-maternal communication and maintenance of pregnancy in mammals (Bazer et al., 2010), as well as the development of preimplantation embryos in cattle (Li et al., 2012). For miR-92b-3p, KEGG pathways of the target genes were mainly enriched in hormone-related pathways, including the Estrogen signaling pathway and GnRH signaling pathway in the top 20 enriched pathways. Notably, estrogen of conceptus origin is the major signal for maternal recognition of pregnancy in pigs, acting in an endocrine manner to prevent luteolysis caused by prostaglandin F2α (PGF2α) and subsequently sustaining progesterone secretion for maintaining the pregnancy (Bazer et al., 1982). This implicated that the increase of circulating miR-92b-3p in pregnant pig may be triggered by early conceptus signals. GnRH, gonadotropin-releasing hormone, is released by the hypothalamus and involved in coordinating the levels of hormones in the hypothalamic-pituitary-gonadal axis, indicating a decisive role in mammalian reproduction. It has been reported that the circulating exosomal miRNAs could pass through the blood–brain barrier and placental barrier to participate in intercellular communication (Ouyang et al., 2014; Smythies et al., 2014). Therefore, we speculate that the increased expression levels of miR-17-5p and miR-92b-3p in maternal serum in early pregnancy could probably be delivered to distal tissues, such as hypothalamus, via circulating exosomes and strengthen the regulation of pregnancy-related factors, such as progestin and estradiol. Further studies are needed to validate the source of these circulating exosomal miRNAs and understand their potential regulatory mechanisms to the target tissues.

## Conclusion

In summary, through RNA sequencing and qPCR profiling in pigs, we for the first time identified alterations in the levels of exosomal miRNAs in serum as early as day 9 of pregnancy. Our results indicate that circulating exosomal miRNAs could probably play important roles in early pig pregnancy through their targeted genes participating in the reproductive pathways. In particular, we identified increased levels of miR-92b-3p and miR-17-5p on days 9–15 of pregnancy. Both miRNAs could be considered as novel biomarkers for differentiating the pregnancy status of pigs as early as day 9 of pregnancy.

## Data Availability Statement

The raw reads produced in this study were deposited in the NCBI Sequence Read Archive (SRA), the records can be accessed by accession number PRJNA577009.

## Ethics Statement

The animal study was reviewed and approved by Ethics Committees of the Laboratory Animal Center of South China Agricultural University (Approval No. SYXK-2014-0136). Written informed consent was obtained from the owners for the participation of their animals in this study.

## Author Contributions

CZ, GC, LH, and ZW designed the study and drafted the manuscript. YH, QH, EZ, SH, ZQX, and TG collected the treatment samples. FM, BH, ZEX, and CZ conducted the experiments and performed the sequencing analysis. All authors read and approved the final manuscript.

## Conflict of Interest

The authors declare that the research was conducted in the absence of any commercial or financial relationships that could be construed as a potential conflict of interest.

## Footnotes

1 http://www.genome.jp/kegg/

2 http://kobas.cbi.pku.edu.cn/
